# The Inhibition of Neuropathic Pain Incited by Nerve Injury and Accompanying Mood Disorders by New Heme Oxygenase-1 Inducers: Mechanisms Implicated

**DOI:** 10.3390/antiox12101859

**Published:** 2023-10-13

**Authors:** Irene Suárez-Rojas, Montse Pérez-Fernández, Xue Bai, Ignacio Martínez-Martel, Sebastiano Intagliata, Valeria Pittalà, Loredana Salerno, Olga Pol

**Affiliations:** 1 Grup de Neurofarmacologia Molecular, Institut d’Investigació Biomèdica Sant Pau (IIB Sant Pau), 08041 Barcelona, Spain; 2 Grup de Neurofarmacologia Molecular, Institut de Neurociències, Universitat Autònoma de Barcelona, 08193 Barcelona, Spain; 3 Department of Drug and Health Sciences, University of Catania, 95125 Catania, Italy; 4 Department of Molecular Medicine, Princess Al Jawhara Centre for Molecular Medicine, College of Medicine and Medical Sciences, Arabian Gulf University, Manama 329, Bahrain

**Keywords:** analgesia, anxiety, depression, dimethyl fumarate, heme oxygenase 1, inflammasome, neuropathic pain, Nrf2 transcription factor, oxidative stress

## Abstract

Neuropathic pain is a type of pain that persists for a long time and becomes pathological. Additionally, the anxiodepressive disorders derived from neuropathic pain are difficult to palliate with the current treatments and need to be resolved. Then, using male mice with neuropathic pain provoked by chronic constriction of the sciatic nerve (CCI), we analyzed and compared the analgesic actions produced by three new heme oxygenase 1 (HO-1) inducers, 1m, 1b, and 1a, with those performed by dimethyl fumarate (DMF). Their impact on the anxiety- and depressive-like comportments and the expression of the inflammasome NLRP3, Nrf2, and some antioxidant enzymes in the dorsal root ganglia (DRG) and amygdala (AMG) were also investigated. Results revealed that the administration of 1m, 1b, and DMF given orally for four days inhibited the allodynia and hyperalgesia caused by CCI, while 1a merely reduced the mechanical allodynia. However, in the first two days of treatment, the antiallodynic effects produced by 1m were higher than those of 1a and DMF, and its antihyperalgesic actions were greater than those produced by 1b, 1a, and DMF, revealing that 1m was the most effective compound. At four days of treatment, all drugs exerted anxiolytic and antidepressant effects, decreased the NLRP3 levels, and increased/normalized the Nrf2, HO-1, and superoxide dismutase 1 levels in DRG and AMG. Data indicated that the dual modulation of the antioxidant and inflammatory pathways produced by these compounds, especially 1m, is a new promising therapeutic approach for neuropathic pain and related emotional illnesses.

## 1. Introduction

Neuropathic pain is a chronic pain triggered by a lesion or sickness of the somatosensory nervous system [1]. There is evidence that both anxiety and depressive disorders are highly associated with the suffering of neuropathic pain [2]. Moreover, a bidirectional relationship has been found between the intensity with which depression and anxiety manifest themselves and the magnitude of the pain complaint [3]. The pharmacological treatment of neuropathic pain is mainly based on the use of anticonvulsants, antidepressants, and opioids, which have limited efficacy and cause multiple side effects [4]. In the search for novel treatments able to reduce the nociceptive and affective symptoms of neuropathic pain, we propose to investigate new heme oxygenase-1 (HO-1) enzyme inducers capable of activating the endogenous pain resolution systems and simultaneously inhibiting the inflammatory pathways involved in the development of neuropathic pain. It is well accepted that chronic sciatic nerve constriction (CCI)-induced neuropathic pain increases reactive oxygen species (ROS) levels, which leads to an imbalance of redox-type reactions, causing oxidative stress [5], mitochondrial dysfunction, and apoptosis [6], the main bases of neurodegenerative diseases [7]. Elevated levels of ROS and oxidative stress biomarkers, such as 3-nitrotyrosine and malondialdehyde, have been demonstrated in the sciatic nerves of animals with CCI-induced neuropathic pain. Additionally, there was a reduction in the levels of antioxidant molecules, including glutathione, superoxide dismutase, and catalase, in these nerves. This reflects the oxidative stress caused by CCI, revealing the increased susceptibility of neurons of the sciatic nerve to hyperalgesia [8,9]. Moreover, the administration of a ROS scavenger, phenyl-N-tert-butylnitrone, improved the thermal and mechanical hypersensitivity generated by nerve injury, verifying the significance of ROS in the maintenance of neuropathic pain [10].

Oxidative stress also induces the expression of numerous inflammatory mediators implicated in the development of neuropathic pain and the associated emotional disorders [11]. One of them is the NLRP3 inflammasome, whose levels were significantly increased in the spinal cord as well as in the hippocampus and prefrontal cortex (PFC) of animals with neuropathy [12,13,14]. Moreover, its inhibition reversed neuropathic pain caused by CCI [15] or associated with multiple sclerosis [16] and also ameliorated the anxiety- and depression-like behaviors associated with type 2 diabetes in mice [17]. Recent studies further demonstrate that some treatments alleviate the allodynia induced by nerve injury by suppressing NLRP3 inflammasome activation in the spinal cord [12,18].

Going deeper into the field of pain and the main molecular bases related to the endogenous antioxidant pathways, a remarkable enzyme participating in this process is HO-1. This enzyme has been demonstrated to possess numerous anti-inflammatory and immunomodulatory properties [19,20] and is under the control of the nuclear factor erythroid 2-related factor 2 (Nrf2). In stressed situations, this transcription factor is liberated from the Keap1/Nrf2 complex and travels to the nucleus, initiating the transcription of multiple antioxidant and detoxifying genes. These genes include, besides HO-1, glutathione S-transferase Mu 1 (GSTM1), superoxide dismutase 1 (SOD-1), and NAD(P)H: quinone oxidoreductase 1 (NQO1) [21]. In consequence, it has been shown that several Nrf2 activators, such as dimethyl fumarate (DMF) [22,23], and HO-1 inducers, for instance, cobalt protoporphyrin IX (CoPP), inhibit neuropathic pain provoked by chemotherapy or sciatic nerve injury [24,25,26], as well as the inflammatory pain provoked by the subplantar administration of complete Freund’s adjuvant (CFA) [27]. In addition, Casili et al. (2020) [28] demonstrated that DMF reduced the rates of anxiety and depression associated with migraine in mice, but these effects during neuropathic pain have not yet been evaluated. Considering that the affective deficits accompanying chronic pain can negatively affect the sensation of pain [29], it is essential to find new compounds that, besides pain, can also inhibit the related emotional symptoms.

**Figure 1:**
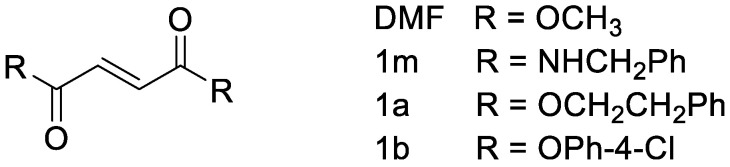
Chemical structure of DMF and HO-1 inducers, 1m, 1a, and 1b, was designed and synthesized by [30,31].

Chemical manipulation of the DMF backbone led to the discovery of novel HO-1 inducers derived from DMF, including 1m, 1b, and 1a [30,31]. New compounds were designed by maintaining the central electrophilic dicarbonyl function of DMF and modifying the methoxy residues with the aim of increasing the volume of the substituents and gaining additional non-covalent interactions at the target (Figure 1). Most potent HO-1 inducers exhibited an unsubstituted benzyl amide function (1m), a phenethyl ester moiety (1a), or a 4-chloro phenyl ester residue (1b).

These new compounds can activate the Nrf2 expression as DMF and induce the HO-1 expression more effectively than DMF itself. Moreover, 1m and 1b reduce ROS production provoked by palmitic acid without cytotoxic activity in cell cultures [30]. Since the potential use of these new compounds as a therapy for neuropathic pain and the accompanying mood disorders has not yet been studied, this will be one of the main objectives of this work. Our hypothesis proposes that the oral administration of 1m, 1b, and/or 1a will inhibit the allodynia and hyperalgesia provoked by a nerve injury with greater effectiveness than DMF. Then, in male mice C57BL/6 with neuropathic pain provoked by CCI, we assessed (1) the actions of the repetitive administration of 1m, 1b, 1a, and DMF given orally on the allodynic and hyperalgesic responses incited by CCI; (2) the possible actions of 1m, 1b, 1a, and DMF on the affective deficits linked with neuropathic pain; and (3) the main molecular mechanisms participating in the actions produced by these treatments.

## 2. Materials and Methods

### 2.1. Animals

We employed male C57BL/6 mice purchased from Envigo Laboratories (Barcelona, Spain), maintained in controlled conditions of temperature, humidity, and light (21 ± 1 °C, 55 ± 10%, 12 h light-dark cycle), with food and water available ad libitum. All animal procedures were approved by the local ethical committee of Animal Use and Care of the Autonomous University of Barcelona (ethical code 9863) and conducted in accordance with the guidelines of the European Communities Council Directive (2010/63/EU) and the Spanish Law (RD 53/2013). Experiments were performed between 9:00 a.m. and 5:00 p.m. and after 7 days of habituation in the animal room. All possible actions were performed to reduce the number of mice and their suffering to the maximum.

### 2.2. Induction of Neuropathic Pain

We used the CCI model proposed by [32] to induce neuropathic pain since it correlates well with the symptomatology presented by patients. Then, to perform the surgery, mice were anesthetized with isoflurane (induction, 3%; surgery, 2%). After checking the animal’s entry into the deep anesthetic plane and with the position of the femur of the right lower leg at 90° with respect to the spine, the hair was shaved, and the connective tissue between the superficial gluteus and biceps femoris was dissected. The sciatic nerve was exposed, and three ligatures were made, 1 mm apart, with silk thread (4/0; T-4522; Laboratorio Aragó, SL, Barcelona, Spain). Finally, the nerve was reintroduced, the muscle layers were closed, and the skin sutures were made. In control groups (SHAM), the identical procedure was conducted, excluding the ligation of the nerve.

### 2.3. Nociceptive Behavioral Responses

Mechanical allodynia was estimated using the filaments of von Frey by positioning the animals in cylinders (Plexiglas; 9 cm/width and 20 cm/tall) on a wire net and following the procedure described by [33]. Briefly, after 1 h of habituation to the test, filaments with bending forces from 0.4 to 3.5 g were applied on the hind legs according to the up–down paradigm. The test commenced with a 0.4 g filament, and the strength of the next filament was enhanced or diminished according to the mice’s responses. The answer threshold was determined with an Excel program (Microsoft Iberia, SRL, Barcelona, Spain) containing the curve fit of the data.

We used the plantar test to measure thermal hyperalgesia [34]. The animals were placed in cylinders (Plexiglas; 9 cm/width and 20 cm/tall) on a glass surface, and after 1 h of habituation to the test, radiant heat was placed under hind legs until the paw withdrawal. A maximum of 12 s was established. Three different measurements, taken 5 min apart, were performed to obtain the paw withdrawal latency (s) of the animals.

To measure thermal allodynia, the response to a cold stimulus using the cold plate test (Ugo Basile, Varese, Italy) was used. In this procedure, each animal was placed on a cold plate (5 ± 0.5 °C), and the sum of each hind paw elevation during 5 min was quantified.

### 2.4. Emotional Behavioral Responses

The test used to assess the anxious-like behaviors was the elevated plus maze (EPM). As indicated by [35], it is equipped with four arms with a length of 35 cm long and 5 cm wide; two of them are open, and the other two are closed by walls 15 cm high. The mice were positioned in the maze center, always facing the same open arm. The total entries to the arms (open and closed) and the time remaining in open arms (%) for 5 min were quantified.

The tail suspension test (TST) and forced swimming test (FST) were employed to assess depressive-related conduct according to the procedures described by [36,37]. In the TST, animals were suspended with an adhesive tape by the tail at a height of 35 cm, and the immobility time (s) was estimated at 6 min. For the FST, animals were introduced into a methacrylate cylinder with water at 24 ± 1 °C and 10 cm of depth. The subjects were filmed for 6 min, and the time that the animal remained immobile (s) was determined during the last 4 min.

All procedures were executed by experimenters blinded to the experiments, and mice were accustomed to the environment for 1 h prior to the tests.

### 2.5. Western Blot

Animals, SHAM and CCI, were euthanized at 28 days of surgery by cervical dislocation without anesthesia. The ipsilateral DRG from the lumbar section (L3 to L5) and the contralateral AMG extracted following Paxinos and Franklin’s stereotaxic coordinates [38] were stored at −80 °C until usage.

Primary antibodies were: anti NLRP3 (1:200; Adipogen Life Sciences, Epalinges, Switzerland); HO-1 (1:150; Enzo Life Sciences, New York, NY, USA); NQO1 (1:250; Sigma-Aldrich, St. Louis, MO, USA); Nrf2 (1:100; Abcam, Cambridge, UK); SOD-1 and GSTM1 (1:150; Novus Biologic, Littleton, CO, USA), and anti-glyceraldehyde-3-phosphate dehydrogenase (GAPDH; 1:5000; Merck, Billerica, MA, USA).

Tissues were sonicated in ice-cold lysis RIPA buffer containing 0.5% and 1% of protease and phosphatase inhibitor cocktail (Sigma-Aldrich, St. Louis, MO, USA) and kept for 1 h at 4 °C. Samples were sonicated again (10 sec) and centrifuged (4 °C; 20 min; 700 g). They were separated on a 12% sodium dodecyl sulfate-polyacrylamide gel and transferred onto a polyvinylidene fluoride membrane for 120 min. Membranes were blocked at room temperature for 1 h with 5% dry milk or 5% bovine serum albumin in phosphate-buffered saline plus Tween 20 or Tris-buffered saline plus Tween 20 for 75 min and incubated at 4 °C overnight with the primary antibody. After washing, membranes were incubated with the secondary antibody (GE Healthcare, Little Chalfont, UK) for 1 h at room temperature, developed using the ECL kit reagents (GE Healthcare, Little Chalfont, UK), detected with the Chemidoc MP system (Bio-Rad, Hercules, CA, USA), and analyzed using the Image-J software version 1.8.0 (National Institutes of Health, Bethesda, MD, USA).

### 2.6. Drugs

DMF was acquired by Sigma-Aldrich (St. Louis, MO, USA). Additionally, 1m, 1b, and 1a were produced by the research group of Prof. Salerno at the University of Catania [30,31]. Compounds were dissolved in 0.05% Cremophor EL/dimethyl sulfoxide/ethanol from Sigma-Aldrich (St. Louis, MO, USA) in a mixture of 12:2:2 in compliance with other work [39]. All compounds were prepared before usage in 10 mL/kg of volume and orally administered. For every group injected with a drug, the corresponding control group was treated with a vehicle (0.05% Cremophor EL/dimethyl sulfoxide/ethanol; VEH).

### 2.7. Experimental Design

Firstly, SHAM and CCI mice were orally administered with 300 mg/kg of 1m (0.1019 mol/L), 1b (0.0889 mol/L), 1a (0.0924 mol/L), DMF (0.2081 mol/L), or VEH for 4 consecutive days, from day 25 to 28 afterward surgery. The doses were chosen in accordance with a study [22], demonstrating that DMF orally administered is effective in reversing the mechanical allodynia caused by nerve injury in mice at 300 mg/kg but not at lower doses. Then, considering the high similarity between DMF and 1m, 1b, or 1a, we chose this dose and the oral route of administration for these drugs.

The evaluation of the tactile and cold allodynia, as well as the thermal hyperalgesia, was performed prior to the injury (day 0) or treatment (day 24 after surgery) and on each day of treatment (from day 25 to 28 after surgery) at 3 h after drug administration in compliance with [39] (*n* = 6 per group, 60 mice in total) and in accordance with the following sequence: von Frey filaments, plantar test, and cold plate test.

The evaluation of the effects of 1m, 1b, 1a, and DMF on the anxious- and depressive-like behaviors linked with neuropathic pain was performed in other groups of SHAM and CCI mice according to the following sequence: EPM, TST, and FST. These animals were also orally administered 300 mg/kg of 1 m, 1b, 1a, or DMF for four consecutive days, from day 25 to 28 after surgery. The assessment of the anxiety- and depressive-like behaviors was conducted on day 28 after surgery (*n* = 8 animals per group, that is, 48 mice for all groups)—in total, 108 mice.

In all the groups studied, one test was started once the previous test had finished, always maintaining the same order of the animals. The impact of treatment with 1m, 1b, 1a, or DMF on the NLRP3 inflammasome, Nrf2, and the enzymes (SOD-1, NQO1, HO-1, and GSTM1) levels in the dorsal root ganglia (DRG) and the amygdala (AMG) in animals with neuropathic pain was evaluated by using Western blot.

### 2.8. Statistical Analyses

The data are plotted as the mean ± standard error of the mean (SEM). The SPSS version 28 (IBM, Madrid, Spain) and GraphPad Prism 9.0 software (La Jolla, CA, USA) programs were employed for the statistical analysis. The assessment of the effects of surgery (SHAM or CCI), treatment (1m, 1b, 1a, DMF or VEH), time of treatment, and their potential interactions was performed by using a three-way analysis of variance (ANOVA) of repetitive measures. For each test and day, a one-way ANOVA and the Tukey test were performed to estimate the possible differences between treatments.

A one-way ANOVA and Tukey tests were employed to assess the plausible variations among 1m, 1b, 1a, DMF, or VEH treatments in modulating anxiety- and depressive-like behaviors. The same test was used to examine the impact of treatments on the expression of different proteins in the DRG and AMG.

A *p* < 0.05 was admitted as significant.

## 3. Results

### 3.1. Effects of 1m, 1b, 1a, and DMF Given Orally on the Hyperalgesia and Allodynia Provoked by Sciatic Nerve Injury

**Figure 2:**
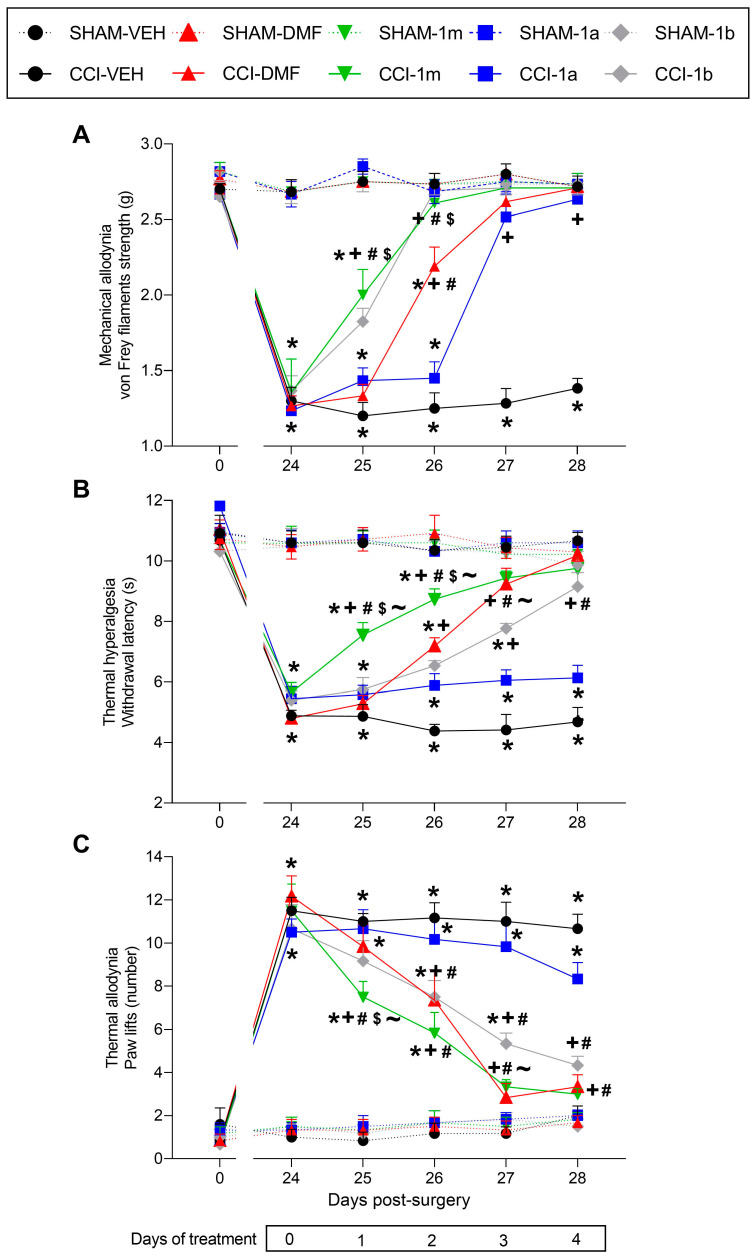
Effects of treatment with 1m, 1b, 1a, and DMF on the mechanical allodynia, thermal hyperalgesia, and thermal allodynia provoked by sciatic nerve injury in mice. The inhibition of mechanical allodynia (**A**), thermal hyperalgesia (**B**), and thermal allodynia (**C**) produced by the repeated oral administration of 1m, 1b, 1a, and DMF from day 25 to 28 after operation is shown. SHAM mice have been employed as controls. In every test, group, and time assessed, symbols represent significant changes: * vs. their corresponding SHAM animals; + vs. CCI-VEH; # vs. CCI-1a; $ vs. CCI-DMF; and ~ vs. CCI-1b (*p* < 0.05, one-way ANOVA followed by the Tukey test). Data are presented as mean values ± SEM (*n* = 6 animals per group).

We assessed the impact of 1m, 1b, 1a, and DMF orally given on sciatic nerve injury-induced mechanical allodynia, thermal hyperalgesia, and thermal allodynia from day 25 to 28 post-surgery (Figure 2). For the mechanical allodynia, significant effects of surgery (F (1,5) = 577.35, *p* < 0.001), treatment (F (4,20) = 50.83, *p* < 0.001), and time (F (5,25) = 61.53, *p* < 0.001) were observed. Additionally, the interaction among surgery and treatment (F (4,20) = 41.49, *p* < 0.001), surgery and time (F (5,25) = 89.37, *p* < 0.001), treatment and time (F (20,100) = 9.60, *p* < 0.001) and among all of them, (F (20,100) = 10.73, *p* < 0.001) were revealed from the three-way ANOVA of repetitive measures.

Our data revealed that the low threshold of the ipsilateral hind paw withdrawal to von Frey filaments provoked by CCI on day 24 from surgery (*p* < 0.001, one-way ANOVA vs. SHAM-VEH) was entirely reversed at four days of treatment with 1m, 1b, 1a, and DMF (Figure 2A, Table 1). Nevertheless, it should be noted that the mechanical allodynia provoked by CCI (*p* < 0.001; one-way ANOVA vs. SHAM-VEH) was entirely inhibited on the second day of treatment with 1m and 1b, while one more day of treatment with DMF and 1a was needed (*p* < 0.001; one-way ANOVA). In addition, the antiallodynic actions of 1m and 1b on the first and second days of treatment were higher than those caused by DMF and 1a on the identical days (*p* < 0.001; one-way ANOVA). Finally, on the second day of treatment, the actions of DMF were higher than those of 1a (*p* < 0.001; one-way ANOVA).

For the thermal hyperalgesia, the three-way ANOVA repeated measures revealed significant effects of surgery (F (1,5) = 663.66, *p* < 0.001), treatment (F (4,20) = 30.39, *p* < 0.001), and time (F (5,25) = 71.01, *p* < 0.001), and of the interaction among surgery and treatment (F (4,20) = 47.04, *p* < 0.001), surgery and time (F (5,25) = 60.02, *p* < 0.001), treatment and time (F (20,100) = 3.93, *p* < 0.001), and among all of them (F (20,100) = 4.97, *p* < 0.001). Results showed that the diminished withdrawal threshold of the ipsilateral hind paw in response to a thermal stimulus observed in CCI animals on day 24 after surgery (*p* < 0.001, one-way ANOVA vs. SHAM-VEH) was completely inhibited after four days of treatment with 1m, 1b, and DMF, but not with 1a (Figure 2B, Table 1). In addition, while three days of treatment with 1m and DMF entirely reverted the thermal hyperalgesia, four days of treatment with 1b were needed. The administration of 1a did not alter the thermal hyperalgesia caused by CCI on any of the days evaluated. Moreover, treatment with 1m stands out for its greater recovery, with significant differences vs. CCI animals treated with DMF, 1b, and 1a on the first and second days of treatment (*p* < 0.001; one-way ANOVA). Lastly, on day three of treatment, the antihyperalgesic effects of DMF were superior to those of 1b and 1a (*p* < 0.001; one-way ANOVA).

Finally, in cold allodynia, the three-way repeated measures ANOVA showed significant actions in surgery (F (1,5) = 299.86, *p* < 0.001), treatment (F (4,20) = 40.46, *p* < 0.001), and time (F (5,25) = 83.66, *p* < 0.001), and of the interaction among surgery and treatment (F (4,20) = 22.08, *p* < 0.001), surgery and time (F (5,25) = 59.07, *p* < 0.001), treatment and time (F (20,100) = 5.47, *p* < 0.001), and among all of them (F (20,100) = 4.52, *p* < 0.001) (Figure 2C, Table 1).

Consequently, the increased number of lifts provoked by cold on day 24 after surgery in the ipsilateral hind paw (*p* < 0.001, one-way ANOVA vs. SHAM-VEH) was entirely reversed on the third day of treatment with 1m and DMF and on the fourth day of treatment with 1b. As occurs for thermal hyperalgesia, the repetitive administration of 1a did not alter the thermal allodynia caused by CCI on any of the days evaluated. In addition, the antiallodynic actions of 1m were greater than those produced by 1b, 1a, or DMF after one day of treatment (*p* < 0.001; one-way ANOVA) and compared to those made by 1a since the second day of treatment (*p* < 0.001; one-way ANOVA). The thermal antiallodynic actions of DMF and 1b were also higher as compared with those performed by 1a from days two to four of treatment (*p* < 0.001; one-way ANOVA).

No variations were found in any test or time evaluated in the ipsilateral paws of SHAM animals (Figure 2) nor in the contralateral paws of CCI or SHAM mice orally given with 1m, 1b, 1a, DMF, or VEH.

### 3.2. Impact of the Administration of 1m, 1b, 1a, and DMF on the Anxiety- and Depressive-Related Conducts Accompanying CCI-Provoked Neuropathy in Mice

**Figure 3:**
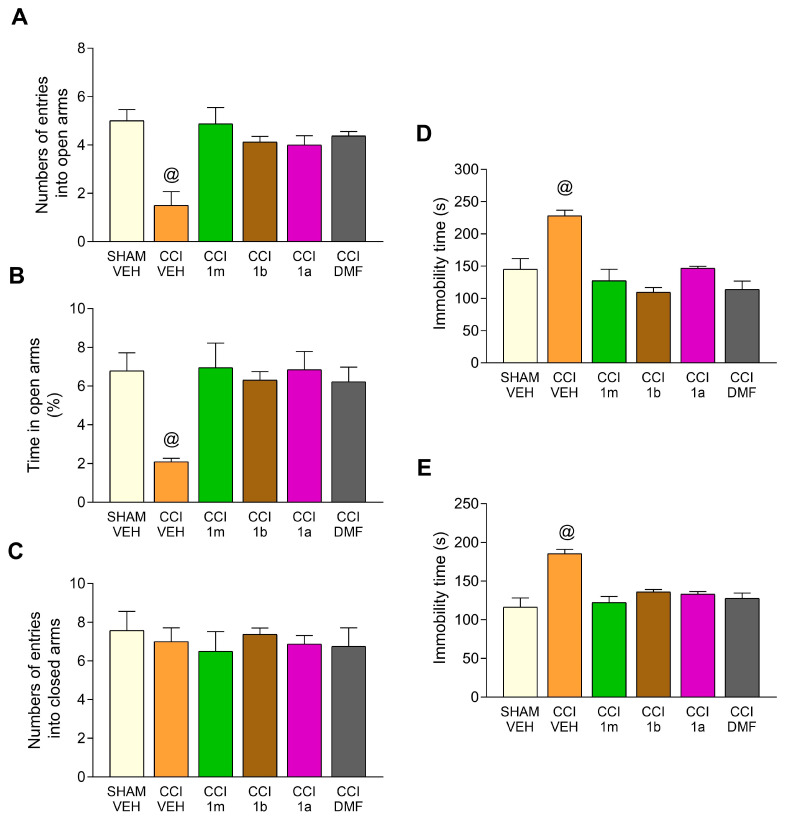
The impact of the therapy with 1m, 1b, 1a, and DMF on the anxiety- and depressive-like behaviors associated with neuropathic pain. In animals orally treated for four consecutive days, from day 25 to 28 after CCI with 300 mg/kg of 1m, 1b, 1a, and DMF, the number of entrances into the open arms (**A**), the percentage of time passed in them (**B**), and the number of entrances into the closed arms (**C**) of the EPM are represented. The immobility times (s) of these animals in the TST (**D**) and FST (**E**) are also displayed. Values from SHAM mice given VEH are furthermore displayed. In all panels, @ indicates significant changes vs. all other groups (*p* < 0.05, one-way ANOVA and Tukey test). Data are presented as mean values ± SEM (*n* = 8 animals per group).

The actions of four consecutive days of treatment with 1m, 1b, 1a, and DMF on the anxious and depressing-like behaviors related to neuropathic pain were evaluated in the EPM, TST, and FST (Figure 3). In the EPM test, all treatments reversed the low number of entries into the open arms (F (5,42) = 8.12, *p* < 0.0001, vs. SHAM-VEH, Figure 3A) and the low percentage of time that CCI animals spent in them (F (5,42) = 5.03, *p* < 0.0001, one-way ANOVA vs. SHAM-VEH, Figure 3B). No changes between groups were observed regarding the number of entrances at closed arms (Figure 3C).

Regarding the depressive-like behaviors, the elevated immobility times of CCI mice injected with VEH in the TST (F (5,42) = 12.59, *p* < 0.001, one-way ANOVA vs. SHAM-VEH, Figure 3D) and the FST (F (5,42) = 12.54, *p* < 0.001, one-way ANOVA vs. SHAM-VEH, Figure 3E) were completely reversed with all treatments.

### 3.3. The Impact of 1m, 1b, 1a, and DMF on the Expression of Inflammatory and Antioxidant Proteins in the DRG and AMG of Animals with Neuropathic Pain

**Figure 4:**
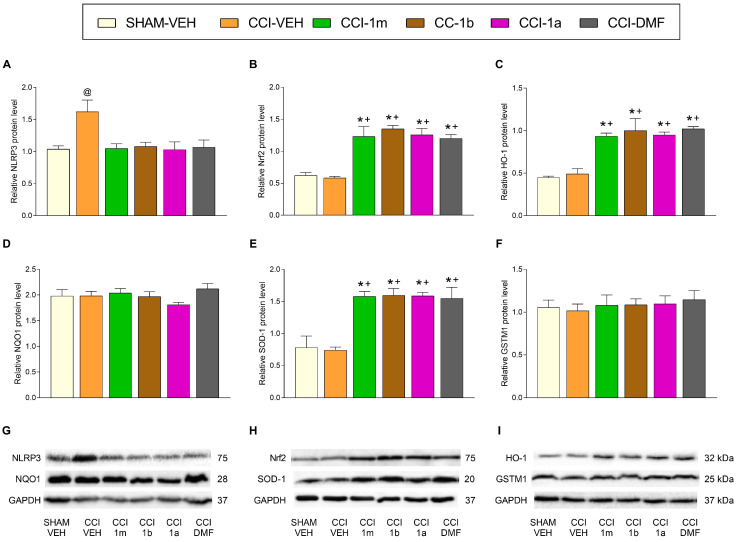
The impact of 1m, 1b, 1a, and DMF on the expression of inflammatory and antioxidant proteins in the DRG of sciatic nerve-injured mice. The effects of 1m, 1b, 1a, and DMF during four days on the NLRP3 (**A**), Nrf2 (**B**), HO-1 (**C**), NQO1 (**D**), SOD-1 (**E**), and GSTM1 (**F**) levels in the DRG of mice at 28 days after surgery are represented. SHAM-VEH-treated animals were used as controls. All proteins are expressed relative to GAPDH protein levels. Representative blots for NLRP3 and NQO1 (**G**), for Nrf2 and SOD-1 (**H**), and for HO-1 and GSTM1 (**I**) are shown. In all figures, * represents significant changes vs. SHAM-VEH, + vs. CCI-VEH, and @ vs. the rest of the groups (*p* < 0.05; one-way ANOVA followed by the Tukey test). Data are presented as mean values ± SEM; *n* = 3 samples.

In the DRG of animals with neuropathic pain treated during four consecutive days with 300 mg/kg of 1m, 1b, 1a, and DMF, one-way ANOVAs indicated that the increased levels of NLRP3 incited by CCI (F (5,12) = 4.41, *p* < 0.016; vs. SHAM-VEH; Figure 4A) were normalized with the administration of each of these treatments. Moreover, the protein levels of NQO1 (Figure 4D) and GSTM1 (Figure 4F) were not altered by any of these treatments. The expression of Nrf2 (F (5,12) = 16.19, *p* < 0.001; Figure 4B), HO-1 (F (5,12) = 15.19, *p* < 0.001; Figure 4C), and SOD-1 (F (5,12) = 12.26, *p* < 0.002; Figure 4E) was significantly increased in animals treated with 1m, 1b, 1a, or DMF as compared to SHAM-VEH and CCI-VEH mice.

**Figure 5:**
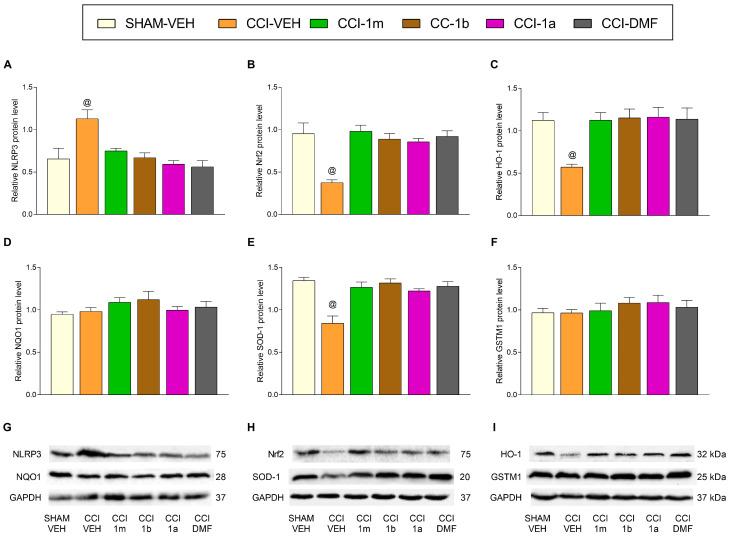
The impact of 1m, 1b, 1a, and DMF on the expression of inflammatory and antioxidant proteins in the AMG of sciatic nerve-injured mice. The effects of treatment with 1m, 1b, 1a, and DMF during four days on the NLRP3 (**A**), Nrf2 (**B**), HO-1 (**C**), NQO1 (**D**), SOD-1 (**E**), and GSTM1 (**F**) levels in the AMG of mice at 28 days after surgery are represented. SHAM-VEH treated animals were used as controls. All proteins are expressed relative to GAPDH protein levels. Representative blots for NLRP3 and NQO1 (**G**), for Nrf2 and SOD-1 (**H**), and for HO-1 and GSTM1 (**I**) are shown. In all panels, @ represents significant differences vs. the rest of the groups (*p* < 0.05; one-way ANOVA and Tukey test). Data are presented as mean values ± SEM; *n* = 3 samples.

In the AMG, one-way ANOVAs indicated that the protein levels of NQO1 (Figure 5D) and GSTM1 (Figure 5F) were not modified by the treatment with 1m, 1b, 1a, and DMF. The increased levels of NLRP3 (F (5,12) = 6.94, *p* < 0.001; Figure 5A) and the decreased expression of Nrf2 (F (5,12) = 9.58, *p* < 0.001; Figure 5B), HO-1 (F (5,12) = 5.50, *p* < 0.007; Figure 5C), and SOD-1 (F (5,12) = 11.35, *p* < 0.003; Figure 5E) provoked by CCI were normalized with all treatments.

## 4. Discussion

This work revealed that the oral administration of 1m, 1b, and DMF for four consecutive days inhibited the mechanical and cold allodynia and thermal hyperalgesia caused by sciatic nerve injury in mice, while 1a only inhibited the mechanical allodynia. All the compounds also had anxiolytic and antidepressant actions, normalized the up-regulation of the NLRP3 inflammasome, and enhanced/normalized the Nrf2, HO-1, and SOD-1 levels in the DRG and AMG of mice with neuropathic pain.

The neuroprotective effects of DMF in neurodegenerative disorders [7], as well as in osteoarthritis pain, post-operative pain, and neuropathic pain caused by spare nerve injury or by chemotherapeutic agents, have been demonstrated [22,23,39,40]. Other studies also revealed that another Nrf2 activator, sulforaphane, as well as the HO-1 inducer, CoPP, both inhibited neuropathic pain provoked by CCI or associated with diabetes [24,25,41,42] and inflammatory pain caused by CFA injection [27]. These studies showed that between three and fourteen days of treatment are required for the complete inhibition of the mechanical allodynia related to neuropathic or inflammatory pain. It is noteworthy that our study demonstrated that a mere two days of treatment with 1m or 1b was sufficient to achieve a complete reversion of the mechanical allodynia provoked by CCI and that their effects were higher than those produced by DMF and 1a. In accordance with [22], three days of treatment with DMF were needed to reverse the allodynia in animals with spared nerve injury-induced neuropathic pain. This reveals the major effectiveness of 1m and 1b in inhibiting CCI-provoked mechanical allodynia. Regarding thermal hyperalgesia, treatment with 1m also produced higher antihyperalgesic actions than those produced by the other drugs in the first two days of treatment. Similarly, the thermal antiallodynic actions of 1m on the first day of treatment were greater than those of the other compounds. In contrast, treatment with 1a did not alter the thermal hyperalgesia or the thermal allodynia provoked by CCI. Thus, this study revealed the major effectiveness of 1m vs. the other compounds in inhibiting neuropathic pain. These results are supported by a detailed structure–activity (SAR) study on a number of similar derivatives towards the molecular target (Nrf2) performed and described [30,31]. With regard to these three molecules (1a, 1b, and 1m), 1m is an amide, which is chemically and enzymatically more stable than 1a and 1b, which are esters. So, we can hypothesize that in vivo, 1m has a longer half-life than 1a and 1b, a pharmacokinetic property that could influence the potency and generally the pharmacodynamics of this compound.

In addition, the augmented levels of Nrf2 and HO-1 observed in the DRG of CCI-animals treated with 1m, 1a, 1b, or DMF might explain the complete inhibition of the mechanical allodynia induced by all of them at four days of treatment. Likewise, all treatments also increased the expression of SOD-1 in the DRG, suggesting the possible involvement of this antioxidant enzyme in their analgesic actions. In this line, the up-regulation of Nrf2, HO-1, and SOD-1 induced by DMF in the DRG of animals with spare nerve injury [22] and enhanced expression of HO-1 stimulated by CoPP in the DRG and sciatic nerves from CCI animals and diabetic mice support our findings [41,42].

It is well acknowledged that Nrf2/HO-1 pathway activation is a critical regulator of cellular stress responses by controlling the redox equilibrium and the inflammatory reactions following nerve injury. The Nrf2 transcription factor may also attenuate the expression of NF-κB, resulting in a negative regulation of the NLRP3 inflammasome activity [43,44]. The NLRP3 inflammasome participates in the development of nerve injury-caused neuropathic pain [45], and its activation can likewise be inhibited by triggering the Nrf2/HO-1 path [44,46]. In accordance with that, our results revealed that the increased expression of NLRP3 in the DRG provoked by CCI was completely reversed by 1m, 1b, 1a, and DMF treatments. These data, together with the activation of Nrf2, HO-1, and SOD-1 induced by 1m, 1b, 1a, and DMF, suggest that these treatments diminished neuropathic pain by a mechanism involving the stimulation of the endogenous antioxidant system and the inactivation of the NLRP3 inflammasome. In accordance, DMF also alleviates dextran sulfate sodium-induced colitis [46], and sulforaphane protects from acute pancreatitis [47] by controlling oxidative stress and the NLRP3 inflammasome through the transcription factor Nrf2. Additionally, other drugs, such as dexmedetomidine, peptide 5, and divanillyl sulfone, improve the allodynia incited by nerve injury by suppressing the NLRP3 inflammasome via Nrf2 activation [12,15,18]. In consequence, the inflammatory and redox equilibrium established by the new DMF derivatives seems to be a promising target for relieving neuropathic pain.

The affective disorders resulting from chronic pain, such as depression and anxiety, are prevalent in patients with neuropathic pain, lowering their quality of life [48]. Although some recent studies have focused on the investigation of the mechanisms underlying affective disorders [49], treatments capable of modulating both pain and emotional symptoms are few. Our results proved the reversion of the anxiogenic- and depressive-like behaviors accompanying neuropathic pain by the repetitive oral treatment with 1m, 1b, 1a, or DMF. These findings revealed, for the first time, the effectiveness of these compounds in inhibiting these emotional disturbances accompanying neuropathic pain as they take place with sulforaphane [50]. Sulforaphane also reduces the depressive-like behaviors linked with stress [51], and other Nrf2 activators, for instance, TBE-31 and MCE-1, or HO-1 inducers, decrease the depressive-like behaviors generated by inflammation [52,53]. Moreover, none of the treatments tested in this work altered the number of accesses to the closed arms in the EPM, revealing the lack of action of 1m, 1b, 1a, and DMF on locomotor activity, thereby reinforcing the safety of these compounds to treat neuropathic pain.

The balance between oxidative and antioxidant responses is crucial to maintaining the homeostasis of the body. Its alteration causes various pathological diseases, such as depression [54]. Consequently, inhibition of oxidative stress can improve mood disorders, and one way to stop oxidative stress is the activation of the endogenous antioxidant system, in which Nrf2 is the main regulator [51]. At the same time, a down-regulation of the Nrf2 pathway can also induce the development of psychiatric diseases. Indeed, a down-regulation of Nrf2 and/or HO-1 was demonstrated in the PFC of patients with mood diseases [55] as well as in the PFC and hippocampus of rodents with anxiodepressive-like conducts related to CCI-induced neuropathy [50]. Furthermore, in agreement with other works [25,56], a down-regulation of Nrf2, HO-1, and SOD-1 in the AMG, a brain area involved in the modulation of emotional responses [57], was revealed in this study. It is important to note that the activity of Nrf2 and its related antioxidant enzymes is down-regulated in anxiodepressive-like states [54]. Moreover, various compounds, particularly activators of Nrf2 and HO-1, are capable of normalizing this negative regulation and suppressing neuropathy-related anxiety-depressive behaviors by regulating oxidative stress [50]. The normalization of the reduced expression of Nrf2, HO-1, and SOD-1 in the AMG of CCI mice produced by 1m, 1a, 1b, or DMF could, at least in part, explain the anxiolytic and antidepressant effects of these treatments during neuropathic pain by regulating the redox homeostasis. In accordance with our findings, the administration of CoPP also normalized the down-regulation of Nrf2, HO-1, and SOD-1 detected in the AMG of diabetic animals with neuropathy [42]. These results support the theory that targeting Nrf2 represents a promising strategy for the treatment of mood disorders [51].

One study by performing morphological analyses demonstrated an increased volume of the AMG in animals with a spared nerve injury accompanied by anxiodepressive behaviors [58]. This alteration was associated with an increased generation of new neurons in the central and basolateral amygdaloid nuclei, while no alterations were found in the dendritic arborizations. These authors also proved that neuropathic pain promotes the generation of new neurons in the AMG and suggested that, given the role of this area in emotive behaviors, these neuroplastic changes might contribute to the development of depressive-like symptoms that are usually present in prolonged pain syndromes in humans [58].

It is well established that neuropathic pain initiates an inflammatory process that, if unresolved, can induce alterations in the normal activity of the CNS [59]. That is, once the peripheral nerve is damaged, immune cells infiltrate at the injury site and initiate the liberation of proinflammatory mediators such as cytokines (TNF-α, IL-1β, and IL-6) and chemokines [60]. Inflammatory signals can be transmitted to supraspinal areas via primary afferent fibers projecting to dorsal horn neurons of the ascending pain pathways, which in turn project to supraspinal regions, including the AMG [61]. Thus, microglial activation has been demonstrated in the AMG of animals with anxiodepressive behaviors associated with CCI-provoked neuropathy [56,62], which takes part in the modulation of these emotive disorders [63]. Barcelon et al., 2019 [62], by assessing gene expression profiles relevant to microglial activation and affective brain dysfunction, found that transcripts of TNF-α were increased in the AMG of animals with affective deficits associated with CCI, which might contribute to its generation [64]. Considering that TNF-α regulates the transcription of the NLRP3 inflammasome [65], the increased expression of NLRP3 observed in the AMG might also be involved in the anxiety and depressive-like behaviors associated with CCI-induced neuropathic pain. In our experiments, the increased expression of NLRP3 in the AMG of CCI animals agrees with the high levels of this inflammasome detected in the hippocampus and PFC of rodents with neuropathic pain [14]. Treatment with 1m, 1a, 1b, or DMF normalized the NLRP3 up-regulation provoked by CCI in the AMG and inhibited the anxiety- and depressive-like behaviors related to neuropathic pain. Thus suggesting that the central anti-inflammatory properties of these compounds might be implicated in their control of affective deficits. In accordance with these findings, the neuroprotective effects of DMF against the depressive-like behaviors associated with chronic unpredictable mild stress [66] and the anxiolytic and antidepressant actions of sulforaphane or luteolin in animals with neuropathic pain [14,50] were also mediated by the activation of Nrf2/HO-1 and inhibition of NLRP3 systems. Finally, and given that MCC950, an NLRP3 inhibitor, reduced the emotional disorders associated with type 2 diabetes in mice [17], our data suggested that the anxiolytic and antidepressant properties of 1m, 1a, 1b, and DMF might in part also produced by blocking the activation of this inflammasome in the AMG. Then, the dual modulation of Nrf2, HO-1, SOD-1, and the NLRP3 pathways may be an interesting therapeutic target for neuropathic pain and linked emotional illnesses.

It is interesting to note that more of the current treatments used in clinical practice for treating neuropathic pain, such as gabapentinoids and several antidepressants [67], can also have an impact on the affective disorders accompanying chronic pain. However, for instance, gabapentin and pregabalin both inhibited the allodynia and the anxiogenic-like behaviors but did not alter the associated depressive-like behaviors [68,69]. On the contrary, several antidepressants, for example, duloxetine, decrease the nociceptive and depressive-like behaviors related to nerve injury that cause neuropathic pain but do not reduce anxiety-related behaviors and induce important side effects [70]. Thus, the high effectiveness of 1m, 1b, and DMF in inhibiting neuropathic pain and both the accompanying affective disorders offers a more global treatment for chronic pain with few side effects.

Finally, neuropathic pain is an important medical problem because of the low effectiveness of conventional therapies and their numerous side effects. The oral administration of compounds like 1m, DMF, and 1b inhibits nociceptive responses caused by CCI and affective behaviors associated with a few days of treatment, especially 1m, making this compound a global, effective, and safe treatment for this type of pain. Furthermore, considering that 1m is a derivative of DMF, which is already used in clinical practice for the treatment of multiple sclerosis [71], a possible clinical trial of 1m in the treatment of neuropathic pain cannot be ruled out.

## 5. Conclusions

In animals with neuropathic pain provoked by CCI, we demonstrated the analgesic effects produced by the repetitive oral administration of 1m, 1b, DMF, and/or 1a, with 1m being the most effective. Additionally, these compounds demonstrate anxiolytic and antidepressant properties by stimulating the endogenous antioxidant system and reducing the inflammatory responses provoked by CCI. In summary, this study proposes the oral administration of 1m as an effective treatment for neuropathic pain and the emotional disorders associated.

## Figures and Tables

**Table 1:** Summary of the results of the one-way ANOVA’s for mechanical allodynia, thermal hyperalgesia, and cold allodynia at 0, 1, 2, 3, and 4 days of treatment with VEH, DMF, 1m, 1a, or 1b in SHAM- and CCI-mice.

Days of Treatment					
	0	1	2	3	4
Mechanical	*F*_9,50_ = 46.38	*F*_9,50_ = 59.07	*F*_9,50_ = 47.26	*F*_9,50_ = 28.31	*F*_9,50_ = 36.74
Allodynia	*p* < 0.001	*p* < 0.001	*p* < 0.001	*p* < 0.001	*p* < 0.001
Thermal	*F*_9,50_ = 49.80	*F*_9,50_ = 49.10	*F*_9,50_ = 37.35	*F*_9,50_ = 30.71	*F*_9,50_ = 26.40
Hyperalgesia	*p* < 0.001	*p* < 0.001	*p* < 0.001	*p* < 0.001	*p* < 0.001
Cold	*F*_9,50_ = 52.36	*F*_9,50_ = 46.45	*F*_9,50_ = 23.81	*F*_9,50_ = 37.03	*F*_9,50_ = 45.64
Allodynia	*p* < 0.001	*p* < 0.001	*p* < 0.001	*p* < 0.001	*p* < 0.001

## Data Availability

Data is contained within the article.
